# Observations on the Hydrocele

**Published:** 1802-07

**Authors:** G. Rowlands

**Affiliations:** Chester


### *Observations on the Hydrocele. Communicated by* Mr. G. Rowlands, *of Chester.*

SEVERAL months ago, a gentleman confulted me about his only fon, a child *five months* old, for a fwelling on one fide of the fcrotum, which not being before obferved, was fuppofed to have come on very fuddenly. It was eafily afcertained to be a collection of fluid in the tunica vaginalis teftis, and I encouraged his parents with the profpect of having it difperfed by the ufe of a lotion.

With this view, compreffes dipped into equal parts of brandy and vinegar, in which a fmall portion of crude fal ammoniac was diffolved, were kept conftantly applied; finding, however, no diminution of the fwelling, the ftrength of the lotion was increafed in the proportion of half an ounce of the falt to fix ounces of brandy and vinegar. This produced much irritation on the fcrotum and uneafinefs to the child, but had no effect on the complaint, for notwithftanding a week's continuance of the application, the tumour ftill became larger, I therefore let out the fluid with the point of a lancet, covered the little orifice with cerate fpread on lint, and repeated the lotion as before. In ten days a fluctuation being again evident, the fluid was a fecond time evacuated, and ftimulating lotions of various kinds were fteadily perfifted in, until all hopes of a cure by that means were at an end.

I was very unwilling to operate in any way for a radical cure, as my little patient was cutting his teeth; but his parents being urgent to have the cure effected before their return to Ireland, I determined to adopt the method by feton, in preference to the incifion, cauftic, or injection, both the incifion and cauftic being at all times very painful in the execution, as well as in the neceffary removal of the dreffings, and, on that account, ought to be rejected; but in the cafe of an infant would be inadmiffible for other reafons, viz. the difficulty or impoffibility of defending the wound from the urine, and of keeping dreffings to the part.

Sir James Earle's eafy and efficacious method by injection, I fhould certainly have employed, as I have invariably done for feveral years, had it been poffible to pierce the hydrocele of fo young a fubject with a trocar, without the utmoft rifk of injuring the tefticle.

The feton, confifting of fix threads, not very fine, was paffed from above downwards, with a common eye probe, which being held in readinefs, I introduced into the puncture the inftant the the lancet was withdrawn; a meaſure neceſſary to be attended to, as much difficulty would enſue in this part of the operation, if the tunica was ſuffered to collapſe.

The following day the ſcrotum ſwelled and inflamed, and gradually increaſed until the third day, when a *little* pus appeared at each orifice. From this time it remained nearly ſtationary for five days, and then began to ſubſide. Two of the threads were now taken away, and the whole of the ſeton on the ninth day from its inſertion.

No other dreſſing was required than ſmall pledgets of ung. lap. calam. over each orifice, which the nurſe was obliged occaſionally to renew.

I believe it has rarely, if ever, been neceſſary to employ any of the operations practiſed for a radical cure of the Hydrocele on ſo young a ſubject, very ſimple external applications, or a ſmall puncture, having proved ſufficient to the cure of the diſeaſe in the ſtate of infancy.

Mr. Pott, when treating of the diſeaſe, ſpeaks of having performed the operation on patients of all ages from *six years* old to ſixty and upwards.

Sir James Earle, Mr. Bell, and others of great experience, who have written on the Hydrocele, make no mention of its having required a radical operation in very young children. And Dr. Underwood, whoſe practice has been extenſive in all the complaints of childhood, ſays poſitively that the ſimple puncture has invariably produced a cure.

I therefore preſume that the recital of this ſolitary caſe, which proved more obſtinate, with the method of treatment, will not be deemed unimportant.

*Chester, May* 28, 1802.

